# An Experimental Study on the Effectiveness and Usefulness of 360° Virtual Reality Simulation in Korean Medical Education: A Pilot Study

**DOI:** 10.3390/healthcare14101426

**Published:** 2026-05-21

**Authors:** Hyun-Kyung Sung, Yongtaek Oh, Mikyung Kim, Eun-Jin Kim, Ju-Hee Lee, Yejin Han, Namin Shin

**Affiliations:** 1 Department of Education, College of Korean Medicine, Dongguk University WISE Campus, Gyeongju 38066, Republic of Korea; shksolar@gmail.com; 2 College of Korean Medicine, Woosuk University, Jeonju 55338, Republic of Korea; ydydxor@gmail.com; 3 Department of Internal Medicine, Dongguk University Ilsan Oriental Hospital, Goyang 10326, Republic of Korea; 01mkkim@gmail.com; 4 Department of Pediatrics of Korean Medicine, Dongguk University Bundang Medical Center, Seongnam 13601, Republic of Korea; utopialimpid@naver.com; 5 College of Korean Medicine, Dongguk University, Goyang 10326, Republic of Korea; jh1548@dongguk.ac.kr; 6 Department of Medical Education & Humanities, College of Medicine, Yeungnam University, Daegu 38541, Republic of Korea; yejinhan@yu.ac.kr; 7 Department of Education, Dongguk University, Seoul 04620, Republic of Korea

**Keywords:** virtual reality, simulation-based education, ultrasound-guided pharmacopuncture, Korean medical education, clinical skills, self-efficacy, usability, pilot study

## Abstract

Background: Virtual reality (VR) simulations provide immersive, interactive learning environments that can support clinical skill development in medical education. However, evidence for its application in Korean medical education remains limited. This pilot study aimed to develop and evaluate HaniE-VR1, a 360° VR simulation program designed to teach ultrasound-guided pharmacopuncture. Methods: A one-group pre–post experimental design was used with 60 undergraduate students from the College of Korean Medicine (pre-intervention *n* = 60; post-intervention *n* = 59, due to one missing post-survey response). The primary outcomes were changes in self-efficacy (MASS) and ultrasound skill-related performance (OSAUS). Secondary outcomes included VR awareness, usability, satisfaction, presence, and cognitive load. Participants completed a VR-based training session using a Meta Quest 3 headset. Effect sizes (Cohen’s d) were calculated for pre–post comparisons. Statistical significance was set at *p* < 0.05. Results: Post-intervention findings showed significant improvements in self-efficacy (MASS: 3.21 ± 0.51 to 3.54 ± 0.61, *p* < 0.001, d = 0.66) and ultrasound skill performance (OSAUS: 2.66 ± 0.73 to 3.54 ± 0.71, *p* < 0.001, d = 1.16). VR awareness also improved significantly (4.33 ± 0.66 to 4.76 ± 0.56, *p* < 0.001, d = 0.65). Participants reported acceptable usability (SUS = 69.49) and high satisfaction (4.51 ± 0.56), confidence (4.32 ± 0.53), and presence (4.40 ± 0.65). Cognitive load and simulator sickness were minimal. Conclusions: The HaniE-VR1 program was associated with improvements in perceived clinical competence, self-efficacy, and learning satisfaction, demonstrating acceptable usability and preliminary educational potential. VR simulations represent a feasible, safe, and engaging approach for integrating experiential learning into Korean medical curricula. Given the exploratory nature of this pilot study, findings should be interpreted with caution, and future controlled research is warranted.

## 1. Introduction

Modern medical education increasingly emphasizes the importance of clinical skills training in enhancing clinical competence. Expanded opportunities to learn and practice diverse clinical skills are associated with greater competence and confidence, which are key factors directly related to patient safety [1,2]. However, skills training using real patients presents several challenges, including concerns regarding patient safety, ethical constraints, infection control, the difficulty of practicing complex procedures, and limited exposure to rare but high-risk clinical situations [2,3,4]. Therefore, simulation-based education has been recognized as an effective and essential alternative to address these limitations [5]. Simulation education offers multiple advantages. First, it enables repeated practice and feedback in a safe learning environment while protecting patients. It also facilitates integrated competency development such as communication, collaboration, and crisis management. Finally, simulation education reduces variability in medical education through standardized scenarios [6,7,8]. Simulation-based medical education has evolved into diverse formats, including low-fidelity task trainers, high-fidelity mannequins, standardized patients, hybrid simulations, and computer-based simulations [9,10,11]. In recent years, the use of immersive technologies such as virtual reality (VR) and augmented reality (AR) in education has rapidly expanded [12]. In particular, VR-based simulation education provides high levels of immersion and interactivity that enhance learner motivation and engagement. VR simulation also allows for repetitive training in a safe, error-tolerant environment, facilitates quantitative tracking of learner performance, and supports individualized feedback and outcome evaluation [13,14,15,16]. Recent evidence has strengthened the educational rationale for VR-based simulations in healthcare education. A recent systematic review focusing on immersive VR in education and training reported that immersive VR environments generally demonstrate positive effects on learning outcomes, particularly when learning objectives and assessment strategies are clearly aligned [17]. In addition, recent umbrella and systematic reviews of medical education have shown that VR- and AR-based interventions are associated with improvements in learner engagement, confidence, and perceived learning effectiveness across a range of healthcare disciplines, emphasizing their role as supplementary educational tools rather than replacements for traditional instruction [18,19]. Comparative reviews have also suggested that VR simulations can achieve learning outcomes comparable to other simulation modalities, although the findings remain sensitive to study design, learner characteristics, and the nature of the targeted skills [20]. Within procedural skills training, VR-based simulations have been reported to yield positive learning effects, including improvements in procedural performance and self-efficacy, across diverse clinical contexts such as emergency medicine, cardiopulmonary resuscitation, surgical skills training, and obstetrics and gynecology education [20,21,22,23]. These findings highlight the growing applicability of VR simulations to complex skill-intensive learning environments. In this context, the present study developed a VR-based simulation training program for ultrasound-guided pharmacopuncture at a Korean medical college. Through a pilot study, the educational effectiveness and usability of the program were evaluated to assess its validity and practical applicability. This study aims to provide preliminary evidence supporting the use of VR technology as a valuable adjunct to clinical procedural skills training in Korean medicine education. The remainder of this paper is organized as follows: Section 2 describes the study design, participants, intervention, and outcome measures; Section 3 presents the results; Section 4 discusses findings and limitations; and Section 5 provides conclusions.

## 2. Materials and Methods

### 2.1. Study Design

This study was designed as a one-group, pre–post pilot study targeting second- to fourth-year undergraduate students at a Korean Medicine College. This study evaluated the initial effects of VR simulation-based education on students’ clinical competency, self-efficacy, learning satisfaction, and discomfort. Of the 60 enrolled participants, 59 completed the post-intervention survey; one participant did not submit the post-survey response and was therefore excluded from pre–post analyses. Given the exploratory, single-group design of this study, observed changes cannot be causally attributed to the VR intervention alone, and findings should be interpreted with caution. The study period was September–October, 2024, and all participants voluntarily consented to participate in the study.

### 2.2. Participants

The study participants were 60 students enrolled in the College of Korean Medicine at D University. The inclusion criteria were students in their second, third, or fourth years of the undergraduate program who were able to understand the study content and sign the consent form. Participants were excluded if they did not agree to the study content or if they experienced severe discomfort, such as motion sickness, from previous head-mounted display (HMD) use. The number of study subjects was calculated using the G*power 3.1.4 program for Windows [24]. For the two-tailed *t*-test, if the effect size (f) was 0.10 or less, it was interpreted as a small effect, 0.25 as a medium effect, and 0.40 or more as a large effect size [25]. Referring to previous studies [26,27], the effect size (d) = 0.50, significance level (α) = 0.05, and power (1 − β) = 0.95 were calculated, and the number of subjects was calculated to be at least 54. Considering a 10% dropout rate, the study aimed to recruit 60 participants. The research participants were recruited through online and offline bulletin board announcements.

### 2.3. Intervention

#### 2.3.1. VR Simulation Program

**Figure 1:**
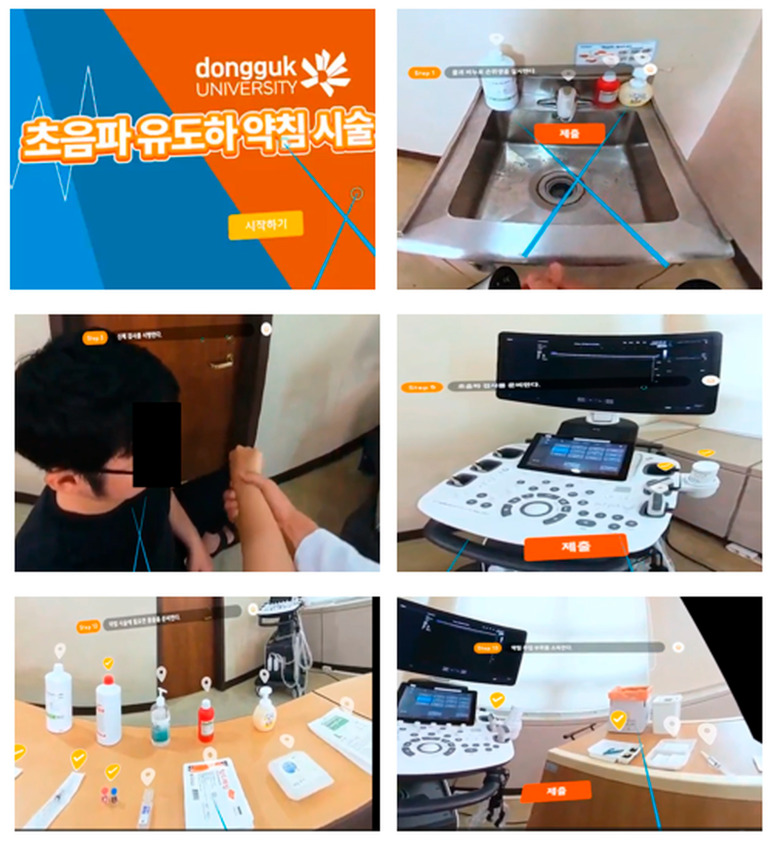
HaniE-VR1 VR simulation program.

The VR simulation training program was designed to teach the process of performing ultrasound-guided pharmacopuncture to patients complaining of shoulder pain. It covers the patient’s medical history, diagnosis, treatment explanation, consent, and the procedure itself. The VR simulation program HaniE-VR1 was developed in collaboration with GlobePoint (Goyang 10550, Republic of Korea), a VR program development company. Based on a clinical scenario, the program featured a standardized patient and a Korean medicine doctor filmed in a real-world medical environment. A 360° VR program was used. Participants wore the Meta Quest 3 HMD (Meta, a product manufactured in CA 94025, USA), used a hand controller and watched the 3D screen (Figure 1). Each step was followed by a quiz, allowing them to experience the process of interacting with, diagnosing, and treating a patient from the perspective of a Korean medicine doctor. The HaniE-VR1 program scenario simulated a patient complaining of shoulder pain visiting a medical institution. It included the following steps: hand washing, self-introduction and confirmation of patient information, physical examination, explanation of ultrasound examination, preparation for ultrasound examination, considerations for ultrasound examination, ultrasound diagnosis, explanation of examination results and treatment plan, preparation for pharmacopuncture, disinfection of the treatment area, pharmacopuncture treatment, and cleanup.

#### 2.3.2. Pilot Experimental Study

Participants received an explanation of the study, signed a consent form, and completed a preliminary survey using Google Forms. A pre-briefing session was then conducted, covering 10–15 min of instruction on how to use the VR device and safety guidelines, as well as the simulation topic of ultrasound diagnosis and pharmacopuncture for shoulder pain. Participants then wore a Meta Quest 3 (including an HMD and haptic controller) and played the HaniE-VR1 VR simulation program for 15–20 min. The participants were encouraged to seek help if they experienced dizziness or other discomfort during the session.

### 2.4. Outcome Measures

Outcome measures were selected to evaluate not only educational effectiveness but also competencies related to patient care, clinician–patient interaction, and clinical decision-making within simulated clinical scenarios.

#### 2.4.1. Subject Characteristics

The questionnaire regarding subject characteristics consisted of questions about the participants’ sex, age, grade, and grades from the previous semester. Based on previous research, it also included questions about their preferred study media [28] and whether they had experienced migraines or motion sickness [29].

#### 2.4.2. Assessment of Understanding and Awareness of VR

Referring to previous research on the understanding and awareness of VR/AR [30], a questionnaire was adapted for VR. Questions on VR understanding were administered before the simulation, whereas awareness was measured before and after the simulation. Understanding consisted of four questions, and awareness consisted of 13 questions, measured on a 6-point Likert scale.

#### 2.4.3. Medical Achievement Self-Efficacy Scale (MASS)

MASS was developed to measure self-efficacy in medical education [31]. It consists of 18 items that were modified and administered on a 5-point Likert scale before and after the program.

#### 2.4.4. Objective Structured Assessment of Ultrasound Skills (OSAUS)

The OSAUS was developed to assess ultrasound-related training. It assesses not only the direct performance of ultrasound equipment but also various other aspects, such as the need for ultrasound examinations and how they contribute to future treatment. This tool was previously modified and adapted for use [32,33]. The items consisted of seven essential sub-items for ultrasound examinations and were administered on a 5-point Likert scale before and after the program.

#### 2.4.5. Usability Test

The usability questionnaire was originally developed to measure the ease of use and usability of the program [34], and was modified and adapted to evaluate the usability of the VR program. The questionnaire consisted of nine questions about the ease of use of the program and seven questions about its usefulness, was administered pre- and post-test, and used a 5-point Likert scale.

#### 2.4.6. System Usability Scale (SUS)

Developed by Brooke (1986), the SUS has been used to evaluate programs in various fields [35,36]. This scale was modified and adapted to evaluate the usability of VR programs. The questionnaire consisted of 10 items and was measured post-test on a 5-point Likert scale. Scores were calculated by subtracting 1 from odd-numbered items and 5 from even-numbered items, calculating the sum of the adjusted scores, and then multiplying by 2.5 to obtain the standard SUS score.

#### 2.4.7. Evaluation of Presence and Educational Effectiveness

A questionnaire from a previous study [37] was used to measure the presence and educational effectiveness of VR programs. The questionnaire consisted of 15 items: five on presence, four on learning effectiveness, and six on learning persistence. The questionnaire was administered as a post-test on a 5-point Likert scale.

#### 2.4.8. Learning Self-Efficacy Scale for Clinical Studies (L-SES)

The L-SES was adapted from a questionnaire developed by Kang et al. [38] based on Bloom’s Taxonomy of Educational Objectives. The questionnaire consisted of 12 items: four cognitive, four affective, and four psychomotor. The questionnaire was administered as a post-test using a 5-point Likert scale.

#### 2.4.9. Student Satisfaction and Self-Confidence in Learning Scale (SCLS)

The SCLS was developed in a previous study by Jeffries et al. [39] and adapted for this study. The questionnaire consisted of five satisfaction items, eight self-confidence items, and ten active learning items. The questionnaire was administered as a post-test using a 5-point Likert scale.

#### 2.4.10. Simulation Design Scale (SDS)

The SDS was developed based on a previous study by Jeffries et al. [39] The questionnaire consisted of 20 questions: five on objectives/information, four on support, five on problem solving, four on feedback/guided reflection, and two on fidelity/realism. The questionnaire was administered as a post-test using a 5-point Likert scale.

#### 2.4.11. Simulation Sickness Questionnaire (SSQ)

The SSQ was developed to assess motion sickness when using simulator systems. Based on the Motion Sickness Questionnaire, which was developed to assess motion sickness caused by transportation, the SSQ was developed to assess simulation-induced discomfort [40,41]. The SSQ is used to measure the degree of motion sickness in VR environments and is particularly used as an indicator of discomfort in studies using HMDs [42,43,44]. The questionnaire was administered as a post-test using a 4-point Likert scale for 16 discomfort items, with higher scores indicating greater discomfort.

#### 2.4.12. NASA-Task Load Index (NASA-TLX)

The NASA-TLX was initially developed as a tool to measure workload in the aviation field [45] and is currently used in assessments across various fields, including transportation, medicine, and computing [46,47,48]. The assessment tool consisted of six subscales: mental demand, physical demand, temporal demand, effort, performance, and frustration. Each item was rated as a post-test on a scale from 1 to 100, with higher scores indicating a greater cognitive load.

#### 2.4.13. AttrakDiff-2 Evaluation

AttrakDiff-2 is a tool for analyzing content usability from both practical and hedonic perspectives [49]. It categorizes evaluation items into four categories: pragmatic quality (PQ), hedonic quality identity (HQ-I), hedonic quality stimulation (HQ-S), and attractiveness (ATT). These were further subdivided into 28 subcategories, allowing for individual and comparative analyses with other categories. The questionnaire consisted of seven items related to practicality, seven items related to identity, seven items related to stimulation, and seven items related to attractiveness, and was measured post-test using a 7-point Likert scale in which respondents chose from −3 points for negative adjectives to 3 points for positive adjectives for each adjective.

### 2.5. Data Collection and Analysis

A self-administered survey was administered to the participants using Google Forms before and after the experiment. Pre–post analyses were conducted on data from the 59 participants who completed both surveys. IBM SPSS Statistics, version 29.0 (IBM Corp., Armonk, NY, USA) was used for data analysis. Descriptive statistical analysis was performed to determine frequencies, means, and standard deviations. Inferential statistical analyses (independent *t*-test, paired *t*-test, and one-way ANOVA) were conducted to examine group differences. Effect sizes were calculated using Cohen’s d based on the standard deviation of paired differences, as computed by SPSS. Values of d ≥ 0.2, ≥0.5, and ≥0.8 were interpreted as small, medium, and large effects, respectively [25]. Statistical significance was set at *p* < 0.05. The primary outcomes were changes in self-efficacy (MASS) and ultrasound skill-related performance (OSAUS). Secondary outcomes included VR awareness, usability, satisfaction, presence, and cognitive load. Given the exploratory nature of this pilot study, no formal correction for multiple comparisons (e.g., Bonferroni adjustment) was applied to the primary and secondary outcome analyses; findings should therefore be interpreted with caution. Post hoc Bonferroni correction was applied to one-way ANOVA subgroup analyses.

### 2.6. Ethical Considerations

This study was approved by the Institutional Review Board of D University Hospital of Korean Medicine (No. DUIOH 2024-08-001-001). All participants provided written informed consent after receiving a thorough explanation of the purpose and procedures of the study. This study adhered to the ethical principles of the Declaration of Helsinki.

## 3. Results

### 3.1. Characteristics of Research Participants

Participants’ characteristics are presented in Table 1. The study included 22 males (36.7%) and 38 females (63.3%). The average participant age was 24.14 ± 1.63 years for males and 24.13 ± 2.31 years for females, for an average of 24.13 ± 2.09 years for the cohort. Twenty-nine participants (48.3%) were second-, 19 (31.7%) were third-, and 12 (20.0%) were fourth-year undergraduate students. When asked about their preferred learning medium, 32 participants (53.3%) answered textbooks, 22 (36.7%) answered lectures, 3 (5.0%) answered drawings, 1 (1.7%) answered flashcards, 1 (1.7%) answered models, 1 (1.7%) answered YouTube, and 0 (0%) answered other. When asked about their usual migraine headaches, 12 participants (20.0%) answered yes, and 48 (80.0%) answered no. When asked about their usual motion sickness, 17 (28.3%) answered yes, and 43 (71.7%) answered no. In response to a question about their understanding of VR, all 60 people (100.0%) had heard of VR, and in response to a question about their experience using VR devices, 18 people (30.0%) had no experience with it, 38 people (63.3%) had used it 0–5 times, 4 people (6.7%) had used it 5–10 times, 0 people (0%) had used it 15–20 times, and 0 people (0%) had used it more than 20 times. In response to a question about whether they knew about the characteristics of VR, such as the differences between VR and AR/MR/XR, three people (5.0%) knew VR well, and 57 people (95.0%) did not. In response to the question about whether they owned a VR device, zero people (0%) said they did and 60 (100%) said they did not.

### 3.2. Results of Results of VR Understanding and Awareness Assessment

**Figure 2:**
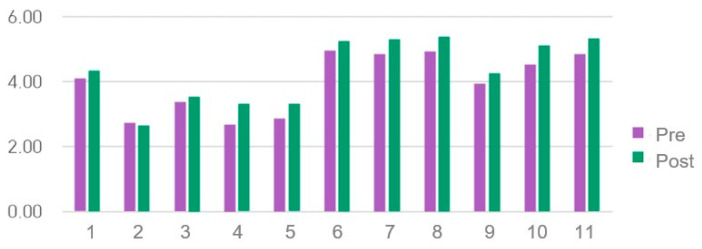
Assessment of understanding and awareness of virtual reality survey results.

The pre- and post-test results of the ‘Assessment of Understanding and Awareness of VR’ survey are shown in Table 2 and Table 3 and Figure 2. The Cronbach’s α in previous studies were 0.88 [29] and 0.83 [50] while in the present study, it was 0.818. The questionnaire showed a significant increase from 4.33 ± 0.66 at pre-test to 4.76 ± 0.56 at post-test (*n* = 59; t = −5.01, *p* < 0.001, d = 0.65, 95% CI [0.37, 0.93]). Item-level analyses showed significant improvements in items reflecting awareness of VR applications in healthcare and intent to engage with VR-based education: item 4 (VR in medical diagnosis/treatment; t = −3.254, *p* = 0.002), item 7 (need for VR content; t = −4.007, *p* < 0.001), item 8 (interest in VR lectures; t = −4.306, *p* < 0.001), item 10 (necessity of VR in Korean medical education; t = −5.228, *p* < 0.001), item 11 (t = −3.382, *p* = 0.001), item 12 (t = −3.779, *p* < 0.001), and item 13 (t = −3.641, *p* = 0.001); Bonferroni corrected (Table 3), while items reflecting passive media exposure (items 1–3) and content purchase intention (item 9) did not reach significance. No significant differences were observed in aggregate VR awareness scores based on sex, grade, or academic performance.

### 3.3. MASS Scores

**Figure 3:**
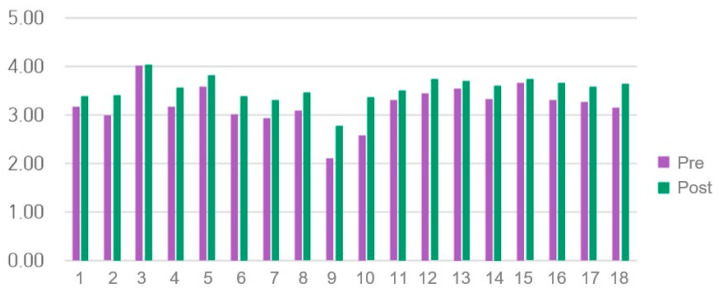
Medical Achievement Self-Efficacy Scale (MASS) results.

The pre- and post-test results using MASS are shown in Table 2 and Table 3 and Figure 3. The Cronbach’s α in the previous study was 0.89 [31], while in the present study, it was 0.874. The mean MASS score significantly increased from 3.21 ± 0.51 before training to 3.54 ± 0.61 after training (*n* = 59; t = −5.084, *p* < 0.001, d = 0.66, 95% CI [0.38, 0.94]). The total score also significantly increased from 57.68 ± 9.17 before training to 63.64 ± 11.03 after training (*n* = 59; t = −5.084, *p* < 0.001, d = 0.662, 95% CI [0.38, 0.94]). No significant differences were observed in MASS scores after training based on sex, grade, or academic performance.

### 3.4. OSAUS Scores

**Figure 4:**
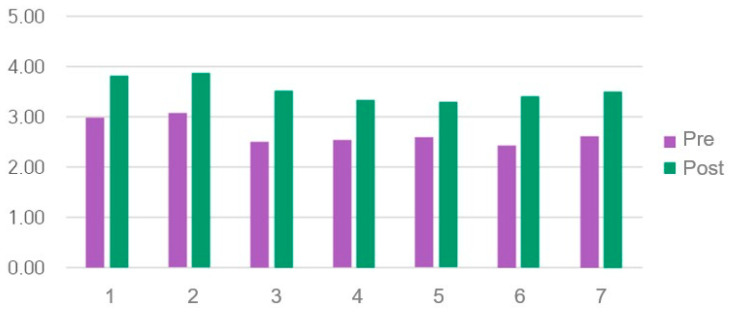
Objective Structured Assessment of Ultrasound Skills (OSAUS) scores.

The pre- and post-OSAUS results are shown in Table 2 and Table 3 and Figure 4. Cronbach’s α in a previous study was 0.916 [51], whereas in this study, it was 0.860. The mean OSAUS score significantly increased from 2.66 ± 0.73 before training to 3.54 ± 0.71 after training (*n* = 59; t = −8.875, *p* < 0.001, d = 1.155), and the total score significantly increased from 18.69 ± 5.16 before training to 24.75 ± 4.97 after training (*n* = 59; t = −8.875, *p* < 0.001, d = 1.155, 95% CI [0.83, 1.48]). No significant differences were observed in OSAUS scores after training based on sex, grade, or academic performance.

### 3.5. Results of Usability Test Scores

**Figure 5:**
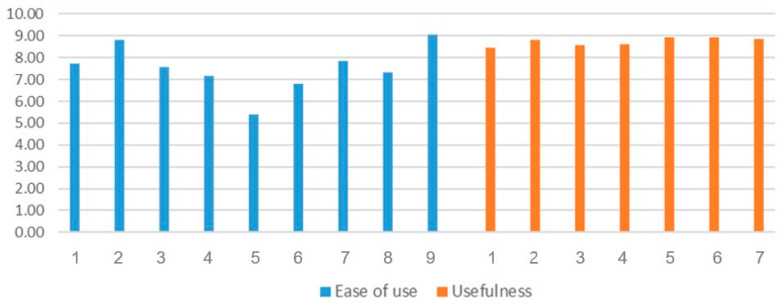
Usability test scores.

The results of the usability tests are presented in Table 4 and Figure 5. In a previous study, Cronbach’s α was 0.080 for ease of use and 0.98 for usefulness [52]. In this study, it was 0.627 for ease of use and 0.947 for usefulness. The post-test results showed no significant differences according to grade. However, women had significantly higher usability scores (8.32 ± 0.89) than men (7.60 ± 1.19; t = −2.664, *p* = 0.010). The mean scores for ease of use were 7.52 ± 1.10, usability was 8.73 ± 1.32, and the overall mean was 8.05 ± 1.06.

### 3.6. SUS Scores

**Figure 6:**
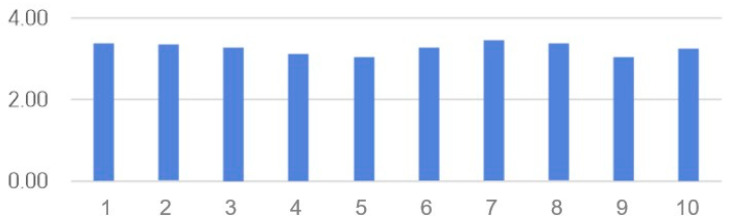
System Usability Scale (SUS) scores.

The SUS results are presented in Table 4 and Figure 6. Cronbach’s α in a previous study was 0.916 [53], whereas in this study, it was 0.761. Generally, a SUS score of 68 or higher is considered above-average [53]. In this study, the SUS score of 69.49 indicated acceptable usability according to established SUS benchmarks, although remaining within the lower range of ‘good usability’ [53]. In the detailed questionnaire, the items “I want to use it often,” “It’s easy to use,” and “I can quickly learn how to use it” all scored high, with scores of 4 or higher, indicating that the program is relatively easy to use. The post-training survey results showed no significant differences by sex, grade, or academic performance.

### 3.7. Reults of Presence and Educational Effectiveness

**Figure 7:**
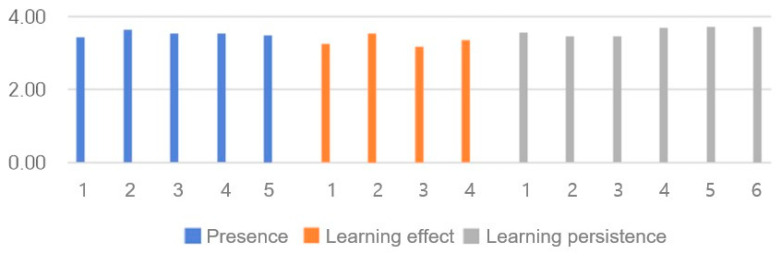
Evaluation of presence and educational effectiveness scores.

The survey results are presented in Table 4 and Figure 7. The Cronbach’s α of the previous study was 0.841 for presence, 0.936 for learning persistence, and 0.945 for learning effectiveness [37]. In this study, the values were 0.881 for presence, 0.901 for learning persistence, and 0.897 for learning effectiveness. The post-education survey results did not show significant differences according to year in school or grades in classes. However, the scores for presence and learning effectiveness were significantly higher in females, with males scoring 4.10 ± 0.70 and females scoring 4.53 ± 0.44 (t = −2.877, *p* = 0.006). The mean score for presence was 4.40 ± 0.65, the mean score for learning effectiveness was 4.16 ± 0.80, and the mean score for learning persistence was 4.49 ± 0.55, with a overall score of 4.37 ± 0.58.

### 3.8. L-SES Scores

**Figure 8:**
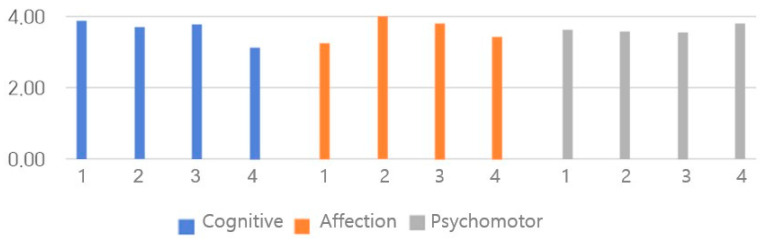
Learning Self-Efficacy Scale for Clinical Studies (L-SES) scores.

The L-SES results are presented in Table 4 and Figure 8. The Cronbach’s α in a previous study was 0.931 [37], whereas in this study, it was 0.910. The post-training questionnaire results showed no significant differences by sex, year in school, or grades in courses. The mean scores were 3.62 ± 0.77 in the cognitive domain, 3.64 ± 0.67 in the affection domain, 3.64 ± 0.71 in the psychomotor domain, and 3.63 ± 0.62 in the total domain.

### 3.9. SCLS Scores

**Figure 9:**
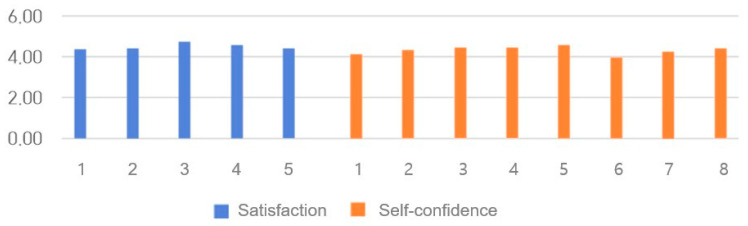
Student Satisfaction and Self-Confidence in Learning Scale (SCLS) scores.

The SCLS results are presented in Table 4 and Figure 9. The Cronbach’s α in a previous study was 0.94 for satisfaction and 0.87 for self-confidence [54]. In this study, satisfaction and self-confidence scores were 0.904 and 0.857, respectively. The post-education questionnaire results showed no significant differences according to year in school or grades in classes. However, female students showed significantly higher learning satisfaction and self-confidence scores, with males scoring 4.21 ± 0.59 and females 4.50 ± 0.44 (t = −2.149, *p* = 0.036). The average scores on the questionnaire were 4.51 ± 0.56 for satisfaction, 4.32 ± 0.53 for self-confidence, and 4.39 ± 0.52 for the total.

### 3.10. SDS Scores

**Figure 10:**
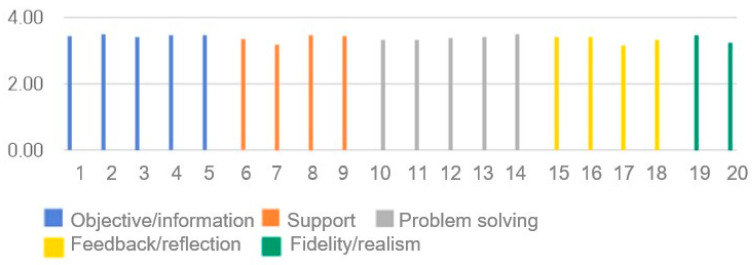
Simulation Design Scale (SDS) scores.

The SDS results are presented in Table 4 and Figure 10. The Cronbach’s α in previous studies was 0.92 [39], while in this study it was 0.943. The results of the post-training questionnaire showed no significant differences according to sex, grade, or academic performance. The average scores for each section were: Objective/Information: 4.31 ± 0.56; Support: 4.19 ± 0.74; Problem Solving: 4.23 ± 0.65; Feedback/Reflection: 4.14 ± 0.75; Fidelity/Realism: 4.19 ± 0.75; and the total average score was 4.22 ± 0.57.

### 3.11. SSQ Scores

**Figure 11:**
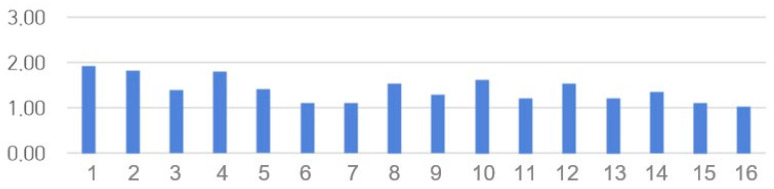
Simulation Sickness Questionnaire (SSQ) scores.

The SSQ results are presented in Table 4 and Figure 11. The Cronbach’s α in a previous study was 0.94 [55], and in this study, it was 0.861. The post-education questionnaire results showed no significant differences by sex or grade. However, SSQ scores were significantly higher in the group with a GPA of 4.0 or higher (1.31 ± 0.35 points) compared to those with a GPA of 3.0 or higher (1.81 ± 0.42 points; F = 2.703, *p* = 0.040). Previous studies have shown that being female or having a history of migraine or motion sickness is associated with higher SSQ scores [55]. However, in this study, sex and the presence of migraines or headaches did not significantly affect the SSQ scores. Scores for all 16 items were below 2.

### 3.12. NASA-TLX Scores

**Figure 12:**
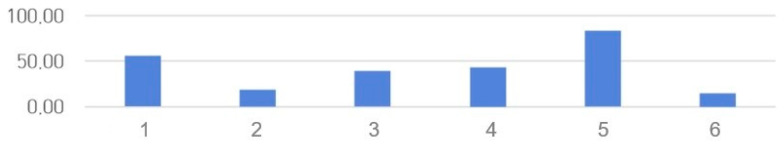
NASA-Task Load Index (NASA-TLX) scores.

The NASA-TLX index was assessed using a 100-point scale. The results are presented in Table 4 and Figure 12. The Cronbach’s α in a previous study was 0.83 [56], while in this study, it was 0.312. However, as the NASA-TLX comprises independent subscales measuring distinct workload dimensions, Cronbach’s alpha is not an appropriate indicator of its reliability and should not be interpreted as a conventional internal consistency estimate [46,57,58]; subscale scores were therefore interpreted individually. The results of the post-education questionnaire showed no significant differences according to sex, grade, or academic performance. The results showed a score of 55.56 ± 23.49 for mental demand, 18.46 ± 18.67 for physical demand, 38.97 ± 23.14 for time demand, 43.42 ± 22.59 for effort, 83.14 ± 94.30 for achievement, and 14.81 ± 14.81 for embarrassment.

### 3.13. AttrakDiff-2

**Figure 13:**
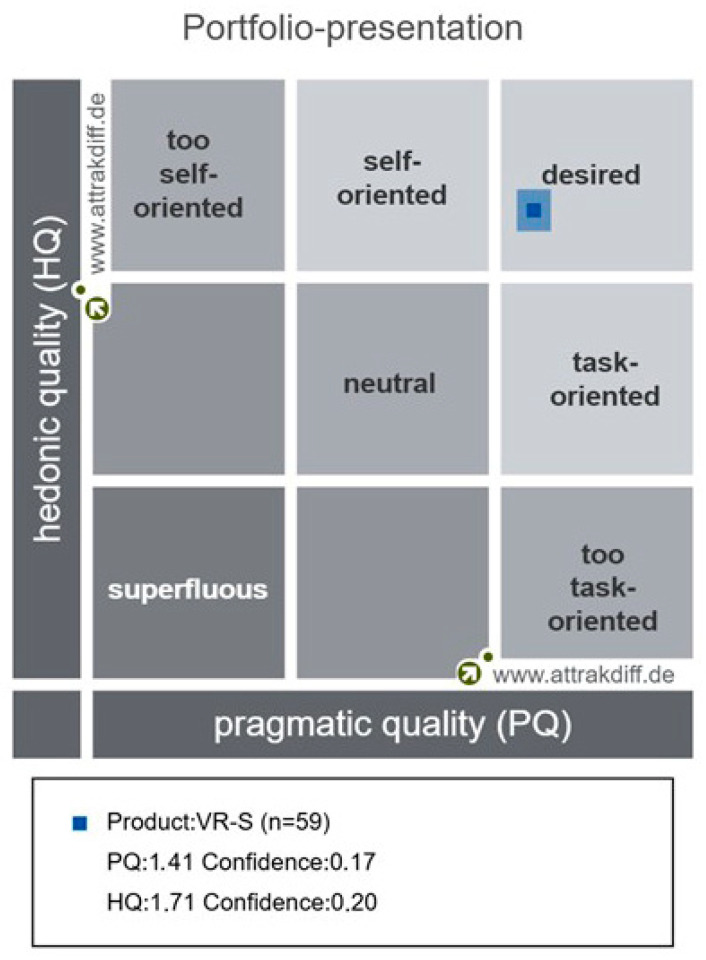
AttrakDiff-2 portfolio presentation.

**Figure 14:**
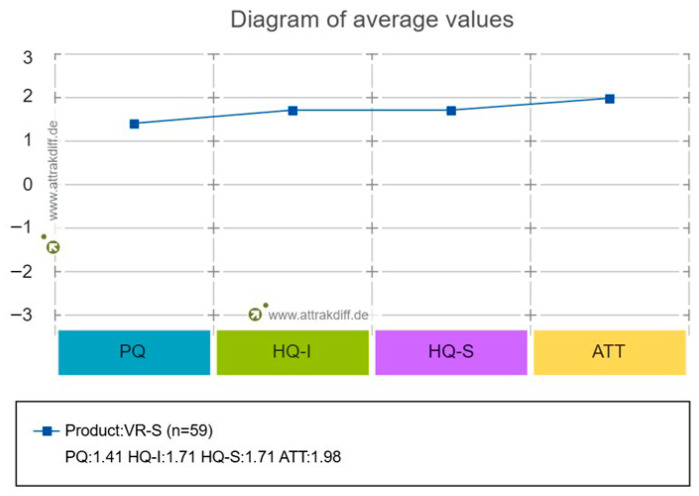
AttrakDiff-2 diagram of average values.

**Figure 15:**
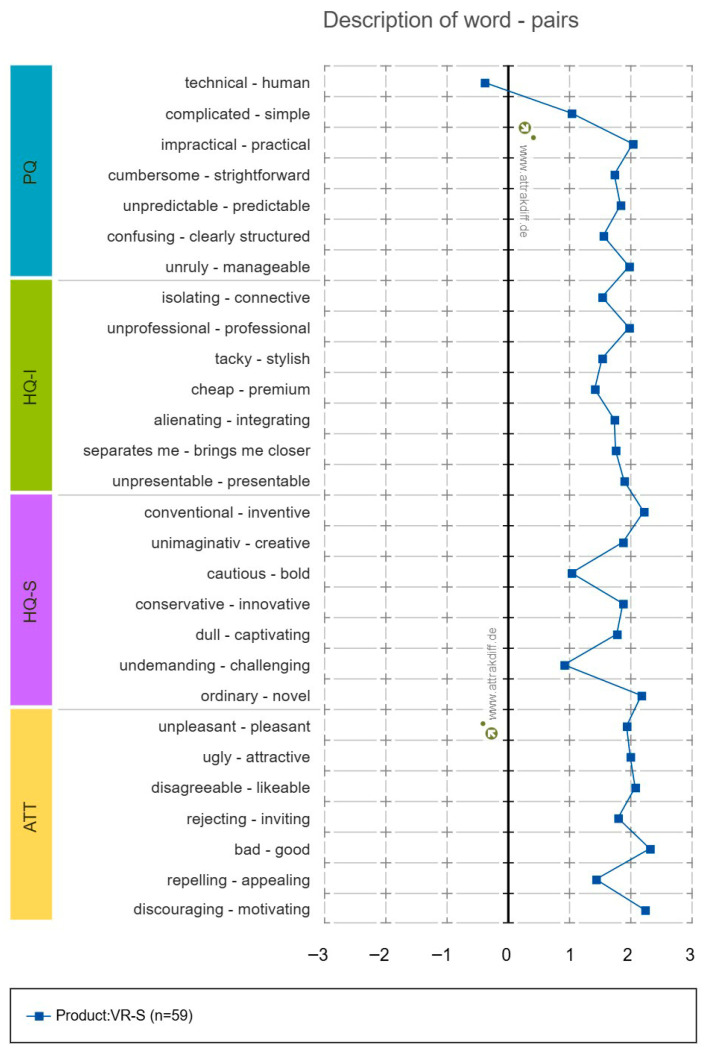
AttrakDiff-2 description of words.

Prior studies on AttrakDiff-2 results showed Cronbach’s α values of 0.62–0.87 for PQ, 0.57–0.67 for HQ-I, 0.76–0.94 for HQ-S, and 0.76–0.93 for ATT [59]. In this study, PQ was 0.596, HQ-I 0.870, HQ-S 0.796, and ATT 0.876. The portfolio presentation results using the analysis program showed that the program’s nature was checked as “desired,” as shown in Figure 13. The results of the analysis of the average values for each part of the diagram are shown in Figure 14, and the results depicting the scores for each word are shown in Figure 15.

## 4. Discussion

This study developed the HaniE-VR1 VR simulation program for clinical practice training in Korean medicine and evaluated its educational effectiveness and usability among 60 enrolled College of Korean Medicine students (59 completing post-intervention assessments) undergoing ultrasound-guided pharmacopuncture training using a single-group pre- and post-test design. The HaniE-VR1 program is a 360° VR simulation based on realistic clinical scenarios designed to replicate the key aspects of real-world clinical practice. Learners wore a Meta Quest 3 (Meta Platforms, Inc., Menlo Park, CA, USA) HMD and used hand controllers to observe three-dimensional clinical scenes and complete step-by-step quizzes, enabling them to experience diagnostic reasoning and treatment decision-making from the perspective of a Korean medicine practitioner. Participants completed a 15–20 min VR session following a 10–15 min pre-briefing. The participant characteristics are summarized in Table 1. Before the intervention, most participants expressed a preference for traditional learning methods, with 53.3% favoring textbooks and 36.7% preferring lectures. Although all participants were aware of the VR technology, active VR usage was relatively limited, with 63.3% reporting fewer than five prior VR experiences. These baseline characteristics suggest that the observed educational effects occurred despite the limited prior familiarity with immersive VR, underscoring the accessibility of the HaniE-VR1 program for novice users. Significant improvements were observed across multiple educational outcome measures following the VR training, including VR awareness, MASS, and OSAUS. Post-intervention VR awareness scores (4.76 ± 0.56) exceeded those reported in previous studies involving nursing students [30], with particularly high gains in perceived benefits, interest, and necessity of VR education. Similarly, the MASS total score increased significantly after a single training session (from 57.68 ± 9.17 to 63.64 ± 11.03, *p* < 0.001, d = 0.662), reaching levels comparable to or exceeding those reported across different medical student grade levels in prior research [31]. These findings suggest that immersive VR simulations may support improvements in learners’ perceived confidence in their clinical abilities, even after brief exposure, although the self-reported nature of these measures warrants cautious interpretation. The total OSAUS score also demonstrated a statistically significant increase following training (from 18.69 ± 5.16 to 24.75 ± 4.97, *p* < 0.001, d = 1.155, 95% CI [0.83, 1.48]), suggesting improved familiarity with ultrasound-related procedures and evaluation criteria. As the OSAUS was administered as a self-assessment instrument in this study, the results reflect learners’ perceived rather than objectively assessed procedural competence. The absence of external evaluators or blinded assessment procedures may have introduced assessment bias. However, the absolute OSAUS scores were generally higher than those reported in a previous validation study [51], where mean scores ranged from 13.6 to 15.4 depending on expertise level. This discrepancy may reflect limited baseline familiarity with the detailed OSAUS scoring criteria among the participants, potentially leading to an overestimation of the initial performance. Importantly, this limitation primarily affects the interpretation of absolute score levels rather than the direction or magnitude of the change. Because identical scoring frameworks and evaluation conditions were applied at both the pre- and post-intervention assessments, the substantial improvement observed was likely to reflect genuine educational gains rather than scoring artifacts. The usability outcomes further support the acceptability of the HaniE-VR1 program. The usability test scores for ease of use, usefulness, and overall usability are consistent with those of previous VR education studies involving nursing and medical students [34,52]. The SUS score of 69.49 exceeded the widely accepted threshold of 68, indicating acceptable usability, although remaining within the lower range of ‘good usability’ according to established benchmarks [53]. Sub-item analyses suggested that while general usability was high, improvements in system stability and visual resolution could further enhance the user experience. The sex-related differences observed in usability and satisfaction scores may reflect differing engagement patterns with immersive technologies and warrant further investigation. The evaluation of presence, learning effectiveness, persistence, self-efficacy, satisfaction, and confidence yielded uniformly high scores comparable to or exceeding those reported in prior simulation-based education studies [60,61,62]. These findings align with existing evidence that VR-based simulation enhances learner engagement, motivation, and perceived educational value. High scores on the SDS further indicated that learners perceived the program as well-structured, realistic, and educationally meaningful. Workload and discomfort assessments indicated that the VR program imposed relatively low physical, temporal, and frustration demands, while eliciting higher mental and performance demands, as measured by the NASA-TLX. As noted in the Results, the NASA-TLX subscale scores were interpreted individually as pragmatic indicators of distinct workload dimensions [46,57,58]. The SSQ scores were uniformly low, suggesting that HaniE-VR1 provides a safe learning environment with minimal VR-related discomfort, which is a frequently cited concern in immersive education research [59,63,64]. The AttrakDiff-2 results further characterized the program as desirable, attractive, and stimulating, supporting its suitability as an educational tool. From an educational perspective, VR simulation programs promote experiential and self-directed learning and have been shown to enhance clinical reasoning, problem solving, and decision-making skills while providing a safe and cost-effective training environment [17,65,66,67,68,69,70,71]. The outcome measures selected in this study were designed to capture not only educational effectiveness but also their relevance to patient care and clinical communication. MASS and OSAUS assessed learners’ perceived confidence in clinical performance and ultrasound-related decision-making, respectively; SCLS reflected self-confidence in clinical procedures; SUS evaluated program usability as a prerequisite for effective learner engagement; and presence measures assessed the realism of simulated clinician–patient interactions. Collectively, these tools provide a multidimensional perspective on the program’s potential to support simulated patient-centered care; however, as all measures were self-reported, they should not be interpreted as direct evidence of objective clinical performance gains. Despite these constraints, HaniE-VR1 provided a positive and engaging learning experience that enhanced satisfaction, confidence, and self-efficacy, which are factors known to contribute to improved learning engagement and perceived clinical readiness [72,73,74,75,76,77,78,79,80,81,82,83,84,85,86,87]. Furthermore, the program exemplifies an integrated and convergent educational approach by combining multiple curricular domains with emerging VR technology, aligning with global trends toward integrated medical education and digital transformation [88,89,90,91,92,93,94,95]. Future iterations incorporating haptic interfaces, artificial intelligence-driven feedback, and objective performance analytics may further strengthen the capacity of VR simulations to support comprehensive clinical skill acquisition.

### 4.1. Limitations

This study has several limitations. First, the study employed a single-group pre–post pilot design without a control group, precluding causal inference regarding the educational effectiveness of VR simulations relative to conventional instruction. Observed improvements cannot be solely attributed to the VR intervention, as maturation, practice effects, and response bias may have contributed. Second, the intervention consisted of a single, short exposure, and outcomes were measured immediately after training. Learning retention, skill transfer, and long-term educational impact could not be evaluated. The novelty effect associated with first-time exposure to immersive VR may have contributed to elevated satisfaction and engagement scores, which may not be sustained with repeated exposure. Third, all outcome measures relied on self-reported data. The OSAUS was administered as a self-assessment instrument, and the absence of blinded external evaluators limits the interpretability of these results as objective measures of procedural competence. Future studies should incorporate blinded, assessor-based evaluation of hands-on skill acquisition. Fourth, generalizability is limited by voluntary participation from a single institution. Participants who voluntarily enrolled may have been more motivated or receptive to VR-based learning than the general student population, potentially introducing selection bias. Fifth, given the exploratory nature of this pilot study, no formal correction for multiple *comparisons* (e.g., *Bonferroni* adjustment) was applied to the primary and secondary pre–post outcome analyses (MASS, OSAUS, VR awareness). This elevates the risk of Type I error and findings should therefore be interpreted with caution. Bonferroni post hoc correction was applied where appropriate in subgroup ANOVA analyses: specifically, the SSQ × GPA one-way ANOVA reached significance (F = 2.703, *p* = 0.040), and Bonferroni correction was applied for pairwise comparisons (GPA 3.0–3.5 < GPA 4.0–4.5). For sex-based independent *t*-tests (Usability, Presence/Educational Effectiveness, SCLS), no post hoc correction was required as each comparison involved only two groups (male vs. female). Grade-level and other GPA-group ANOVAs were all non-significant (all *p* > 0.09); therefore no post hoc correction was needed. Future confirmatory studies should prespecify primary outcomes and apply appropriate statistical corrections a priori. Sixth, interpretation of the OSAUS scores warrants caution. Although a statistically significant improvement in the total OSAUS score was observed, the absolute scores were generally higher than those reported in previous studies. This may reflect limited initial familiarity with the detailed OSAUS scoring criteria among the participants, potentially leading to an overestimation of the baseline performance. In the absence of extensive assessor calibration, blinded evaluations, or external expert raters, some degree of scoring inaccuracy cannot be excluded. Nevertheless, because the same scoring framework and evaluative conditions were applied consistently in both pre- and post-intervention assessments, this limitation is unlikely to invalidate the observed pre-to-post improvement, although it may have influenced the absolute score levels. Additional limitations include the lack of a direct assessment of hands-on skill execution, which is particularly relevant for procedural training, and the limited generalizability of the findings owing to the voluntary participation of students from a single institution. Learners who opted to participate in the study may have been more motivated or receptive to VR-based learning than the general population. Finally, technical constraints inherent in current VR systems restrict the full implementation of all planned procedural elements within scenarios, which may have influenced learners’ perceived realism and training experience.

### 4.2. Future Research Directions

First, the development of VR simulation programs should be grounded in a clearly defined educational framework from the earliest stages, systematically addressing the learning objectives, instructional design, implementation, and outcome evaluation. Future research should explore the standardization of VR simulation training methods and examine the program fidelity, usability, and effectiveness across different VR devices and delivery formats. Additionally, assessment strategies should incorporate standardized performance-based evaluation tools and rater calibration procedures to enhance the validity and interpretability of skill assessments. Second, the unique characteristics of VR technology should be actively integrated into the design and production of VR simulation programs. While most current VR research primarily emphasizes visual and auditory stimuli, further investigation is needed to determine the appropriateness and educational impact of these elements as well as additional interactive features. In particular, future studies should prioritize the integration of haptic interfaces and interactive feedback systems to support objective training and assessment of psychomotor skills that cannot be fully addressed using 360° video-based VR formats alone. Close collaboration with VR technology experts throughout the development process is essential to align educational goals with technical feasibility. Third, a structured pre-training orientation and post-training debriefing should be considered essential components of VR-based education. Although this study observed a relatively low resistance among voluntarily participating students, the implementation of VR in formal curricula may elicit unfamiliarity, discomfort, and resistance among learners. Given that HMD-based VR can induce visual discomfort, dizziness, or motion sickness depending on individual characteristics and exposure duration, future research should examine the optimal training duration, acclimatization strategies, and debriefing approaches. Longitudinal and repeated-exposure study designs are also required to evaluate learning retention, skill transfer, and long-term educational impact. Fourth, objective and comprehensive evaluation frameworks are required to assess the educational effectiveness of VR simulation programs. While this study relied primarily on self-reported questionnaires, future research should combine subjective evaluations with objective performance metrics such as blinded expert ratings, standardized skill assessments, or real-world clinical performance indicators. Controlled study designs with randomized assignments, larger sample sizes, and repeated measurements are critical to disentangling the specific educational contributions of VR-based simulation and establishing its effectiveness relative to conventional instructional approaches. Finally, given the limited body of VR-related research in Korean medical education, further studies are warranted to explore the applicability, benefits, and limitations of VR technology in this context. Accumulating empirical evidence across diverse educational settings and learner populations is essential for developing tailored VR-based educational programs that align with the unique curricular and clinical characteristics of Korean medical education.

## 5. Conclusions

This pilot study suggests that the 360° VR simulation program for ultrasound-guided pharmacopuncture training is a feasible and acceptable educational approach for Korean medical students. The participants demonstrated improvements in perceived procedural competence and self-efficacy, along with positive evaluations of usability and learning experience, indicating that immersive VR simulations may contribute to learners’ perceived readiness for patient-centered care and clinical communication within simulated settings. These findings are consistent with those of previous studies on VR- and simulation-based education, which reported that immersive learning environments can enhance learner engagement, confidence, and perceived learning outcomes when used as a supplementary instructional modality rather than as a replacement for traditional training [58,96]. Prior research has also emphasized the value of VR simulations in providing safe, repeatable, and learner-centered training experiences, particularly for procedural and skill-based education [58,96]. Extending this body of literature, this study applied a 360° immersive VR simulation approach to ultrasound-guided pharmacopuncture training in Korean medical education, an area in which empirical VR-based educational research remains limited. The favorable learner responses observed in this pilot study support earlier findings that immersive VR may be effectively adapted to diverse domains of healthcare education while maintaining usability and learner acceptance [57,58,96]. Nevertheless, considering the exploratory nature of this single-group pilot study, the results should be interpreted with caution. Further research employing controlled and longitudinal designs is required to compare VR-based simulation training with conventional instructional methods, assess its impact on objective clinical performance, and determine its long-term educational value within formal curricula. Despite these limitations, the present findings provide preliminary evidence supporting the feasibility and educational potential of immersive VR simulations in Korean medical education.

## Figures and Tables

**Table 1:** Characteristics of participants.

Category	Subcategory	Number (%)	
Sex	Male	22 (36.67)	
	Female	38 (63.33)	
Average age (years)	Male	24.14 ± 1.63	
	Female	24.13 ± 2.31	
	Total	24.13 ± 2.09	
Grade	2nd grade	29 (48.33)	
	3rd grade	19 (31.67)	
	4th grade	12 (20.00)	
Preferred learning method	Textbook	32 (53.33)	
	Lecture	22 (36.67)	
	Drawing	3 (5.0)	
	Flashcard	1 (1.67)	
	Models	1 (1.67)	
	YouTube	1 (1.67)	
	Other	0 (0)	
Migraines	Yes	12 (20.0)	
	No	48 (80.0)	
Motion sickness	Yes	17 (28.33)	
	No	43 (71.67)	
Understanding of VR	Previous awareness of VR	Yes	60 (100)
		No	0 (0)
	Previous experience with VR	None	18 (30.0)
		0–5 times	38 (63.3)
		5–10 times	4 (6.67)
		15–20 times	0 (0)
		Over 20 times	0 (0)
	Awareness of VR features (differences between VR, AR, MR, XR)	Know	3 (5.0)
		Don’t know	57 (95.0)
	Ownership of a VR device	Yes	0 (0)
		No	60 (100)
Total		60 (100)	

**Table 2:** Pre–post comparison of primary and secondary outcomes with effect sizes (*n* = 59).

Measure	Pre (M ± SD, *n* = 60)	Post (M ± SD, *n* = 59)	t	df	*p*	d	95% CI
Primary Outcomes							
MASS (per-item avg, /5)	3.21 ± 0.51	3.54 ± 0.61	−5.084	58	<0.001	0.662	[0.38, 0.94]
MASS (total score)	57.68 ± 9.17	63.64 ± 11.03	−5.084	58	<0.001	0.662	[0.38, 0.94]
OSAUS (per-item avg, /5)	2.66 ± 0.73	3.54 ± 0.71	−8.875	58	<0.001	1.155	[0.83, 1.48]
OSAUS (total score)	18.69 ± 5.16	24.75 ± 4.97	−8.875	58	<0.001	1.155	[0.83, 1.48]
Secondary Outcome							
VR Awareness (total, /6)	4.34 ± 0.66	4.76 ± 0.56	−5.01	58	<0.001	0.65	[0.37, 0.93]

Cohen’s d: small ≥ 0.2, medium ≥ 0.5, large ≥ 0.8; CI = confidence interval for Cohen’s d.

**Table 3:** Item-level pre–post results: VR Awareness Scale, MASS, and OSAUS (*n* = 59).

	Pre	Post
1. VR awareness		
(1) I have read about VR through various media (internet, articles, news).	4.08 ± 1.32	4.34 ± 1.01
(2) I find information about VR difficult to understand.	2.72 ± 0.96	2.64 ± 0.92
(3) I’ve seen VR used in close environments.	3.37 ± 1.47	3.54 ± 1.22
(4) I know that VR is being used in medical settings for diagnosis or treatment.	2.68 ± 1.30	3.31 ± 1.43
(5) I know VR is being used in medical education.	2.87 ± 1.37	3.31 ± 1.55
(6) I think VR has benefits for education.	4.95 ± 0.87	5.25 ± 0.76
(7) I think there is a need for increased VR content.	4.85 ± 0.90	5.31 ± 0.70
(8) I am interested in attending a VR lecture.	4.93 ± 0.86	5.39 ± 0.72
(9) I am interested in purchasing VR educational content.	3.93 ± 1.19	4.25 ± 1.24
(10) I think that Korean medical education using VR is necessary.	4.53 ± 1.04	5.03 ± 1.03
(11) I am interested in participating in Korean medical education using VR.	4.85 ± 1.04	5.32 ± 0.66
(12) Training based on VR technology will improve clinical skills.	4.90 ± 0.95	5.34 ± 0.68
(13) I think clinical education using VR should be applied.	4.80 ± 0.99	5.31 ± 0.86
Total average	4.33 ± 0.66	4.76 ± 0.56 ***
2. MASS		
(1) I can perform the techniques I have learned so far on patients.	3.17 ± 0.94	3.39 ± 0.95
(2) I have a good insight into the social factors that influence my patients’ health problems.	2.98 ± 0.83	3.41 ± 0.97
(3) I can electronically search the literature related to health issues.	4.02 ± 0.79	4.03 ± 0.77
(4) I can appropriately apply sequential steps of diagnosis and treatment to clinical problems.	3.17 ± 0.79	3.56 ± 0.93
(5) I can respond in an appropriate communication manner in a conflict situation with a patient.	3.58 ± 0.89	3.81 ± 0.80
(6) I am familiar with the medical aspects covered in liberal arts or medical humanities.	3.02 ± 0.97	3.39 ± 1.07
(7) I have sufficient knowledge of basic medicine.	2.93 ± 0.84	3.31 ± 0.86
(8) I can analyze the health problems of patients within a group.	3.08 ± 0.74	3.46 ± 0.90
(9) I can write solid scientific papers on health-related topics.	2.10 ± 1.05	2.78 ± 1.10
(10) I can select/deduce scientific settings to solve medical research problems.	2.58 ± 0.94	3.36 ± 0.92
(11) I can take a personal perspective on the ethical aspects when a patient requests euthanasia.	3.30 ± 0.85	3.51 ± 0.82
(12) I feel I can collaborate on an equal footing with medical professionals from other fields.	3.45 ± 1.00	3.73 ± 1.08
(13) I can manage my emotions when anxiety arises in certain clinical situations.	3.55 ± 0.79	3.69 ± 0.86
(14) I can proactively address health issues in society.	3.33 ± 0.91	3.61 ± 0.81
(15) I can structure the information I get from patients during consultations.	3.67 ± 0.83	3.73 ± 0.85
(16) I can make cost-effective choices when using technological means for diagnosis or treatment.	3.30 ± 1.03	3.66 ± 0.84
(17) I can recognize the symptoms and signs of burnout in my professional role.	3.27 ± 0.94	3.58 ± 0.93
(18) I can handle critical issues (unexpected, stressful events) when practicing medicine.	3.15 ± 0.89	3.64 ± 0.92
Total average	3.21 ± 0.51	3.54 ± 0.61 ***
Total score	57.68 ± 9.17	63.64 ± 11.03 ***
3. OSAUS		
(1) Indication for the examination	2.98 ± 0.91	3.81 ± 0.73
(2) Applied knowledge of ultrasound equipment	3.07 ± 0.94	3.86 ± 0.75
(3) Image optimization: gain, depth, focus, frequency	2.50 ± 0.93	3.53 ± 0.92
(4) Systemic examination	2.53 ± 1.05	3.34 ± 1.08
(5) Interpretation of images	2.58 ± 0.96	3.29 ± 1.00
(6) Documentation of examination	2.43 ± 1.03	3.41 ± 1.15
(7) Medical decision making	2.62 ± 1.11	3.51 ± 1.07
Total average	2.66 ± 0.73	3.54 ± 0.71 ***
Total score	18.69 ± 5.16	24.75 ± 4.97 ***

*** *p* < 0.001.

**Table 4:** Post-survey results.

Question	Result
1. Usability test	
a. Ease of use	
(1) How convenient do you think VR training programs are?	7.73 ± 1.38
(2) Was the initial training on VR devices and how to use them adequate?	8.80 ± 1.42
(3) Was the text information displayed on the VR screen easy to read?	7.56 ± 2.48
(4) Do you clearly understand the video information on the VR screen?	7.17 ± 2.22
(5) Was the resolution of the VR screen good?	5.41 ± 2.69
(6) Have you encountered any difficulties due to errors occurring during VR playback?	6.80 ± 3.13
(7) Was the VR program at an appropriate pace?	7.86 ± 2.05
(8) Was the information displayed on the VR screen appropriately positioned? (Is it consistent and easy to see?)	7.32 ± 2.33
(9) Was the VR device easy to use?	9.03 ± 1.27
Total average	7.52 ± 1.10
b. Usefulness	
(10) Did the on-screen and text information in the VR training program help you learn clinical skills?	8.44 ± 1.51
(11) Did you enjoy the VR training program?	8.81 ± 1.54
(12) Do you expect that your clinical skills will improve through VR training programs?	8.56 ± 1.52
(13) Does the VR training program help you understand clinical skills better?	8.56 ± 1.57
(14) Would you recommend the VR Clinical Skills Training Program to others?	8.93 ± 1.47
(15) Do you think VR training programs will be useful for clinical skills training in the future?	8.93 ± 1.45
(16) Would you consider using VR training programs for learning other clinical skills in the future?	8.86 ± 1.51
Total average	8.73 ± 1.32
Grand total average	8.05 ± 1.06
2. System usability scale (SUS)	
(1) I would like to use this VR simulation training often.	4.22 ± 0.70
(2) I think this VR simulation training is too complicated.	1.97 ± 0.83
(3) I think this VR simulation training is easy to use.	4.10 ± 0.82
(4) I think I need help using this VR simulation training.	3.63 ± 0.95
(5) I think this VR simulation training has a lot of well-integrated features.	3.80 ± 0.98
(6) I feel like there are too many inconsistencies in this VR simulation training.	2.08 ± 0.84
(7) I think most people will quickly learn how to use this VR simulation training.	4.31 ± 0.59
(8) I find this VR simulation training very cumbersome to use.	2.03 ± 0.93
(9) I felt very confident after using this VR simulation training.	3.80 ± 0.76
(10) I had to learn a lot before using this VR simulation training.	2.71 ± 1.18
Total score	69.49
3. Evaluation of presence and educational effectiveness	
a. Presence	
(1) During the VR simulation training experience, I felt as if I was in a clinical setting.	4.27 ± 0.83
(2) I think that this screen, where you experience VR simulation training, is a reality that could actually exist.	4.54 ± 0.68
(3) The sounds (conversations) heard while experiencing VR simulation training seemed real.	4.41 ± 0.77
(4) While experiencing VR simulation training, I felt like I was seeing the scenes on the screen as if they were actually happening.	4.41 ± 0.75
(5) While experiencing the VR simulation training, the situation felt like an actual clinical situation.	4.36 ± 0.92
Total average	4.40 ± 0.65
b. Learning effectiveness	
(1) I feel that my clinical skills have improved more than I expected while learning VR simulation training.	4.05 ± 0.99
(2) I believe that if I study VR simulation training diligently, it will help improve my clinical skills.	4.42 ± 0.72
(3) After receiving VR simulation training, I gained confidence in performing clinical skills.	3.97 ± 1.02
(4) After receiving VR simulation training, it helped me improve my clinical skills.	4.19 ± 0.86
Total average	4.16 ± 0.80
c. Learning persistence	
(1) I plan to use what I learned in the VR simulation training in my future clinical practice.	4.44 ± 0.62
(2) I think I will be able to utilize what I learned in the VR simulation training more in my clinical practice than I expected.	4.31 ± 0.82
(3) I will use what I learned in the VR simulation training in my future clinical practice.	4.31 ± 0.75
(4) I would be willing to experience VR simulation training again.	4.61 ± 0.62
(5) I would recommend VR simulation training to other students.	4.63 ± 0.67
(6) In the future, I would like to utilize VR simulation training more if possible.	4.64 ± 0.52
Total average	4.49 ± 0.55
Grand total average	4.37 ± 0.58
4. Learning self-efficacy scale for clinical scale (L-SES)	
a. Cognitive domain	
(1) I remember how to perform clinical techniques.	3.88 ± 0.85
(2) I can understand the content of clinical techniques and demonstrate them to others.	3.69 ± 0.93
(3) I can verbally explain the purpose and principles of performing clinical techniques.	3.78 ± 1.02
(4) I can verbally explain the sequence and interrelationships between each step of clinical technique.	3.14 ± 0.71
Total average	3.62 ± 0.77
b. Affection domain	
(5) I spend more time on this area than on any other.	3.25 ± 0.96
(6) I think I can get more out of this field than any other.	4.03 ± 0.81
(7) I tend to pay more attention to information related to this field.	3.81 ± 0.80
(8) I actively seek out information related to this field.	3.44 ± 0.90
Total average	3.64 ± 0.67
c. Psychomotor domain	
(9) I can accurately follow the instructor’s steps and actions for clinical techniques.	3.63 ± 0.90
(10) I can smoothly complete the steps of performing clinical skills.	3.58 ± 0.97
(11) I try to monitor my clinical skills for improvement.	3.56 ± 0.84
(12) I strive to monitor clinical skill performance and make appropriate adjustments when necessary.	3.80 ± 0.77
Total average	3.64 ± 0.71
Grand total average	3.63 ± 0.62
5. Student satisfaction and self-confidence in learning scale (SCLS)	
a. Satisfaction	
(1) The teaching methods used in VR simulation training were helpful and effective.	4.37 ± 0.69
(2) VR simulation training provided a variety of learning materials and activities that helped me learn my clinical skills.	4.42 ± 0.70
(3) The VR simulation training was interesting.	4.75 ± 0.51
(4) The learning materials in the VR simulation training were motivating and helpful for learning.	4.58 ± 0.59
(5) The VR simulation training method suited the way I learn.	4.41 ± 0.75
Total average	4.51 ± 0.56
b. Self-confidence	
(1) I am confident that I have learned the contents of the VR simulation training.	4.14 ± 0.78
(2) I am confident that VR simulation training contains important content necessary for learning clinical skills.	4.32 ± 0.84
(3) I am confident that VR simulation training will allow me to develop the skills and gain the knowledge necessary to perform tasks required in a clinical setting.	4.44 ± 0.62
(4) VR simulation training used materials that helped with teaching.	4.44 ± 0.60
(5) It is my role as a learner to learn what I need to know in VR simulation training.	4.58 ± 0.59
(6) I know how to get help when you don’t understand the concepts covered in VR simulation training.	3.97 ± 1.00
(7) I know how to use VR simulation training to learn important aspects of clinical skills.	4.27 ± 0.78
(8) It is the instructor’s role to inform students of what they need to know through VR simulation training.	4.41 ± 0.65
Total average	4.32 ± 0.53
Grand total average	4.39 ± 0.52
6. Simulation design scale (SDS)	
a. Objectives & information	
(1) When starting the VR simulation, sufficient information was available to provide direction and encouragement for learning.	4.29 ± 0.70
(2) Clearly understand the purpose and goals of VR simulation.	4.36 ± 0.61
(3) The VR simulation clearly provided sufficient information in problem-solving situations.	4.25 ± 0.69
(4) I was given enough information during the VR simulation.	4.32 ± 0.71
(5) The instructions within the VR simulation were appropriate and helped enhance my understanding.	4.32 ± 0.80
Total average	4.31 ± 0.56
b. Support	
(6) Support was provided when needed during VR simulation learning.	4.17 ± 0.87
(7) I realized I needed help during my VR simulation learning.	3.98 ± 1.11
(8) I felt supported and assisted by the instructor during the VR simulation.	4.31 ± 0.78
(9) I received support during the VR simulation learning process.	4.29 ± 0.77
Total average	4.19 ± 0.74
c. Problem solving	
(10) Independent problem solving was promoted after the VR simulation.	4.15 ± 0.81
(11) I was encouraged to explore all the possibilities of VR simulation.	4.15 ± 0.89
(12) The VR simulation was designed to suit my specific level of knowledge and skills.	4.22 ± 0.79
(13) VR simulations provided an opportunity to learn about diagnostic and treatment priorities.	4.25 ± 0.76
(14) VR simulations gave patients the opportunity to set goals.	4.36 ± 0.74
Total average	4.23 ± 0.65
d. Feedback/reflection	
(15) The feedback provided was helpful.	4.24 ± 0.80
(16) Feedback was provided in a timely manner.	4.25 ± 0.80
(17) VR simulations allowed me to analyze my own actions and movements.	3.93 ± 1.10
(18) After the VR simulation, I was able to improve my knowledge level by receiving guidance and feedback from the instructor.	4.15 ± 0.83
Total average	4.14 ± 0.75
e. Fidelity/realism	
(19) The scenario was similar to a real situation.	4.34 ± 0.73
(20) Real-world elements, situations, and variables are incorporated into VR simulation scenarios.	4.05 ± 0.94
Total average	4.19 ± 0.75
Grand total average	4.22 ± 0.57
7. NASA-TLX	
(1) Mental Demand	55.56 ± 23.49
(2) Physical Demand	18.46 ± 18.67
(3) Temporal demand	38.97 ± 23.14
(4) Effort	43.42 ± 22.59
(5) Performance	83.14 ± 94.30
(6) Frustration	14.81 ± 14.81
Total average	42.39 ± 20.37
8. Simulation sickness questionnaire (SSQ)	
(1) General discomfort	1.93 ± 0.79
(2) Fatigue	1.83 ± 0.91
(3) Headache	1.39 ± 0.64
(4) Eyestrain	1.80 ± 0.92
(5) Difficulty focusing	1.41 ± 0.65
(6) Increased salivation	1.10 ± 0.31
(7) Sweating	1.10 ± 0.40
(8) Nausea	1.54 ± 0.93
(9) Difficulty concentrating	1.29 ± 0.53
(10) Fullness of head	1.63 ± 0.87
(11) Blurred vision	1.22 ± 0.53
(12) Dizziness (eyes open)	1.54 ± 0.77
(13) Dizziness (eyes closed)	1.22 ± 0.49
(14) Vertigo	1.36 ± 0.69
(15) Stomach awareness	1.12 ± 0.33
(16) Burping	1.03 ± 0.18
Total average	1.41 ± 0.38

## Data Availability

The data that support the findings of this study are not publicly available due to ethical and privacy restrictions. This study was conducted with undergraduate students at a single institution and approved by the Institutional Review Board of D University Hospital of Korean Medicine (No. DUIOH 2024-08-001-001). Participants provided written informed consent for participation in the study; however, consent for public data sharing was not obtained. Public disclosure of the raw dataset could compromise participant confidentiality, as the data contain demographic and academic performance information that may be identifiable within the small study population (n = 60). Data may be made available from the corresponding author upon reasonable request, subject to institutional and ethical approval.
